# High prevalence of frailty in end-stage renal disease

**DOI:** 10.1007/s11255-016-1306-z

**Published:** 2016-05-10

**Authors:** Diederik Drost, Annette Kalf, Nils Vogtlander, Barbara C. van Munster

**Affiliations:** Department of Geriatrics, Gelre Hospitals, Apeldoorn, The Netherlands; Division of Nephrology, Department of Medicine, Gelre Hospitals, Apeldoorn, The Netherlands; Department of Medicine, University Medical Center Groningen, Groningen, The Netherlands

**Keywords:** Elderly, End-stage renal disease, Frailty, Frailty index, Frailty phenotype

## Abstract

**Purpose:**

Prognosis of the increasing number of elderly patients with end-stage renal disease (ESRD) is poor with high risk of functional decline and mortality. Frailty seems to be a good predictor for those patients that will not benefit from dialysis. Varying prevalences between populations are probably related to the instrument used. The aim of this study was to measure the prevalence of frailty among ESRD patients with two different validated instruments.

**Methods:**

This cross-sectional study was conducted among patients, aged ≥18 years, receiving hemodialysis, peritoneal dialysis and pre-dialysis care between September 2013 and December 2013 in a single dialysis center in Apeldoorn, the Netherlands. Frailty was measured with the frailty index (FI) and frailty phenotype (FP).

**Results:**

Prevalence of frailty by the FI was 36.8 % among 95 participants with ESRD (age: 65.2 years, SD ± 12.0). Frailty prevalence among participants aged ≥65 and <65 years was 43.6 and 27.5 %, respectively. Female sex [odds ratio (OR) 3.3, 95 % confidence interval (CI) 1.3–8.0] and a Charlson comorbidity index score of ≥5 (OR 2.6, 95 % CI 1.0–6.6) were associated with frailty. The FI identified different but overlapping participants as frail compared with the FP; 62.5 % of frail participants according to FI were also frail according to the FP.

**Conclusions:**

Prevalence of frailty among young and elderly ESRD patients is high; being female and having more comorbidity was associated with frailty. Use of a broader definition of frailty, like the FI, gives a higher estimation of prevalence among ESRD patients compared with a physical frailty assessment.

**Electronic supplementary material:**

The online version of this article (doi:10.1007/s11255-016-1306-z) contains supplementary material, which is available to authorized users.

## Introduction

With population aging and improved medical care, there is an increasing number of elderly patients with end-stage renal disease (ESRD) who become dialysis dependent [1, 2]. The prognosis of some of these older patients with ESRD after initiating dialysis is poor; mortality is high and they are at high risk of functional decline [3]. Recently, the concept of frailty, the state of low homeostatic reserve leading to a high vulnerability for sudden adverse health changes, emerged as a possible good predictor of prognosis in the ESRD population [4]. Frail patients undergoing hemodialysis had a 2.6 times higher risk of mortality and 1.4 times higher risk of hospitalization, independent of age, gender, comorbidity and disability, compared with patients who were not frail [4].

However, there is no clear consensus about the exact definition of frailty [5], and multiple frailty assessment instruments are being implemented to identify frail patients [6]. To estimate the prevalence of frailty in databases of ESRD populations, researchers used different modifications for measurement of the criteria of the frailty phenotype model [7]. These modifications, besides possible differences in study populations, have led to diverse results; prevalences varied from 24 % (*n* = 188) to more than two-third of an ESRD population (*n* = 2275) [8–10].

Frailty, measured by the original criteria of the frailty phenotype in a hemodialysis population, was found in all ages with an overall prevalence of 42 % [4]. Compared to the prevalence of 7 % in a community-dwelling population of ≥65-year elderly [7], it is clear that, although varying prevalences are found, prevalence of frailty in the ESRD population is high.

Therefore, given the large differences of frailty prevalence, it is important to identify whether disagreement in frailty measurements plays a role. The aim of this study is to establish the prevalence of frailty in ESRD patients measured with two validated assessment instruments: the *Frailty Index* [11] (FI) and the *Frailty Phenotype* [7] (FP) in one population and to determine whether the risk factors for frailty from the literature correspond to the risk factors in an ESRD population.

## Subjects and methods

### Study population

This cross-sectional study was conducted in a single dialysis center in Gelre Hospitals in Apeldoorn, the Netherlands, from September 2013 till December 2013 in patients aged 18 years and above. Patients receiving chronic hemodialysis, chronic peritoneal dialysis and patients in pre-dialysis care were asked to participate. Pre-dialysis care group consists of patients with an estimated glomerular filtration rate (eGFR) of <20 ml/min/1.73 m^2^ but did not yet need renal replacement therapy and those patients who are expected to be renal replacement therapy dependent quickly, due to fast decline of kidney function. The study was reviewed by the Medical Ethics Committee (METC) of the Academic Medical Center Amsterdam (Reference Number W13_164 # 13.17.0209). Written informed consent was obtained from participants.

### Data collection

At enrollment, trained research staff collected medical information from the medical charts, including diagnosed comorbidities according to the Charlson comorbidity index [12]. eGFR was calculated using the 4-variable Modification of Diet in Renal Disease (MDRD) study formula [13]. Participants were asked to complete a questionnaire and performed a set of function tests supervised by trained research staff (Table S1, online-only). Maximal grip strength (average of three measurements) was measured in the dominant hand with a type 5030J1 Jamar hydraulic dynamometer. Walking speed (normal and rapid pace) was measured as the fastest time of two measurements [7, 14]. Unrecordable grip strength and inability to walk were scored as positive items on both frailty instruments.

### Frailty

Frailty was established according to the FI [11] and the FP [7]. We used 38 variables and cutoff points as used by Searle et al. [14], consisting of physical, psychological, social and cognitive items, and documented comorbidity, excluding shoulder strength and peak flow measurement (Table S1, online-only). The FI was the total deficits as a proportion of those counted. The FI was graded in three categories equivalent to the FP. A FI of ≤0.08 was considered as non-frail, a FI of >0.08 and <0.25 as pre-frail and a FI of ≥0.25 as frail [15]. The five components of the FP were measured: weight loss, exhaustion, weakness, slow walking speed and physical activity (Table S1, online-only).

### Statistical analysis

Descriptive statistics were given for all baseline demographic and clinical data. Baseline characteristics were compared among frail and non-frail patients (according to dichotomized FI). Normally distributed, non-normally distributed continuous variables and nominal variables were tested with a Student’s *T* test, Mann–Whitney *U* test or Chi-square test, respectively. All patient characteristics variables with *p* value of <0.10 or associated with frailty based on the existing literature [5] were included in a multivariable logistic regression analysis with the dichotomized FI as outcome. Participants were excluded from analysis of an instrument if they had missing values for more than 20 % per instrument [16]. The statistical significance level was set to 0.05. All analyses were performed using Statistical Package for Social Sciences (SPSS) software version 20.

## Results

### Study population

Of a total of 144 patients, 95 (66 %) participated in this study. Individuals in the included and non-responders group were similar with respect to age, treatment modality and comorbidity index. In the included group, there was a lower proportion of females compared to the non-responders group (43 vs. 68 %; *p* = .004). The mean age of the 95 participants was 65.2 years [Standard Deviation (SD) ±12.0], range 27 to 88 years. Forty-two participants (44 %) were undergoing hemodialysis, 14 (15 %) were undergoing peritoneal dialysis, and 39 (41 %) were receiving pre-dialysis care.

### Frailty

Of the total study population, 36.8 % were considered frail by the FI. Frailty prevalence was 43.6 % in participants of 65 years and above and was 27.5 % in the younger group. Women were more likely to be frail compared to men (51 % of female vs. 26 % of male; *p* = .01) (Table 1). No difference was found in age between frail (mean 66.6, SD ± 13.5; *p* = .39) and non-frail (mean 64.4, SD ± 11.1) individuals; correction for gender or treatment modality did not alter this result. Frail participants had a (one point) higher Charlson comorbidity index score (median: 4, IQR 3–6), compared to non-frail participants (median: 3, IQR 2–5; *p* = .019). Treatment modality did not differ between the frail and non-frail participants.

**Table 1:** Patient characteristics: frailty status, according to frailty index [11]

Characteristic	Non-frail (*n* = 60)	Frail (*n* = 35)	*p* value
Female (%)	20 (33.3)	21 (60.0)	.01
Age (years)	64.4 (±11.1)	66.6 (±13.5)	.39
Race, Caucasian (%)	58 (96.7)	33 (94.3)	.42
Treatment modality (%)			
HD	25 (41.7)	17 (48.6)	.80
PD	9 (14.5)	5 (15.2)	
Pre-dialysis	26 (43.3)	13 (37.1)	
Body mass index	27.1 [24.0–29.8]	27.0 [25.0–29.4]	.81
Laboratory			
eGFR (mL/min/1.73 m^2^) (non-dialysis, *n* = 39)	14.0 [13.0–16.0]	16.0 [13.5–20.5]	.10
Albumin (g/dL)	3.49 (±.36)	3.33 (±.53)	.10
Hemoglobin (g/dL)	11.47 (±1.21)	10.94 (±1.55)	.07
Urea nitrogen (mg/dL)	61.64 [50.40–69.44]	52.23 [39.87–74.69]	.12
Time on dialysis, months (*n* = 56)	14.0 [8.8–43.3]	8.0 [4.5–18.8]	.06
No dialysis (%)	26 (43.3)	13 (37.1)	.21
<12 months	14 (23.3)	14 (40.0)	
≥12 months	20 (33.3)	8 (22.9)	
MMSE (*n* = 87)	28.5 [27.0–29.0]	27.0 [26.0–29.0]	.15
Number of hospitalization last year	1 [0–2]	1 [0–3]	.45
Number of medication	9.0 [8.0–13.8]	12.0 [10.0–14.0]	.03
Comorbidity Charlson index score	3 [2–5]	4 [3–6]	.02

Data are presented as: number (%), mean (±SD: standard deviation) or median [25–75 % IQR interquartile range]

*HD* hemodialysis, *PD* peritoneal dialysis, *eGFR* estimated glomerular filtration rate, *MMSE* mini mental state examination. Conversion factors for units: serum albumin in g/dL–g/L, ×10; serum hemoglobin in g/dL–mmol/L, ×0.6206; urea nitrogen in mg/dL–mmol/L, ×0.357

### Predictors of frailty

Age was not associated with frailty [odds ratio (OR) 2.0, 95 % CI 0.9–4.9] (Table 2). In the multivariable analysis, female sex (OR 3.3, 95 % CI 1.3–8.0) and a Charlson comorbidity index score of ≥5 (OR 2.6, 95 % CI 1.0–6.6) were associated with frailty (Table 2).

**Table 2:** Regression analysis of variables associated with frailty

Variable	Univariate		Multivariate	
	OR (95 % CI)	*p* value	OR (95 % CI)	*p* value
Age (≥65 years)	2.0 (0.9–4.9)	.11		
Female sex	3.0 (1.3–7.1)	.01	3.3 (1.3–8.0)	.009
Hemoglobin (g/dL)	0.6 (0.4–1.1)	.08		
Albumin (g/dL)	0.9 (0.8–1.0)	.07		
Time on dialysis				
No dialysis	1 [reference]			
<12.5 months	2.0 (0.7–5.4)	.17		
≥12.5 months	0.8 (0.3–2.3)	.68		
Comorbidity Charlson index score ≥5	2.3 (1.0–5.6)	.06	2.6 (1.0–6.6)	.04

### Frailty assessment

A frailty status was established for all participants according to the frailty index, however, in seven of the 95 participants (7.4 %); no frailty status according to the frailty phenotype could be established because of >20 % missing data.

Prevalence of frailty in this population according to the FI was 36.8 %, compared with a prevalence of 27.3 % according to the FP. Sixty-four of the 88 participants (73 %) were categorized in the same phenotype category (Table 3). Different, but an equal number of participants [49 (56 %)] were found pre-frail by the FI and FP. Twenty (23 %) pre-frail and frail participants according to the FI were categorized by the FP in the lower non-frail and pre-frail category.

**Table 3:** Comparison of frailty assessment instruments

	Frailty index		
	Non-frail (*n* = 7)	Pre-frail (*n* = 49)	Frail (*n* = 32)
Frailty phenotype			
Non-frail (*n* = 15)	7	8	0
Pre-frail (*n* = 49)	0	37	12
Frail (*n* = 24)	0	4	20

Frailty phenotype compared to frailty index

## Discussion

In this cross-sectional study of adults undergoing hemodialysis, peritoneal dialysis or pre-dialysis care, the prevalence of frailty was 36.8 %. Being a female and/or having more comorbidity was associated with frailty, whereas older age was not associated. The frailty index has a lower cutoff point for the definition of frailty as more patients were identified as frail, in comparison with the frailty phenotype in this dialysis population.

To our knowledge, this is the first study that measured a frailty index in a ESRD population. The prevalence of frailty in this population is much higher than the prevalence in community-dwelling elderly [15]. Our findings of a higher prevalence are consistent with findings of studies where frailty was assessed according to the frailty phenotype [4, 8–10]. Several studies used approximations or substitutions for several items of the frailty phenotype. The only study that used the exact same measurements to measure the frailty phenotype developed by Fried et al. found a slightly higher estimated prevalence of 41.8 % among hemodialysis patients, compared with the current study [4]. A possible explanation for this difference could be that our study population consisted of an almost entirely Caucasian population versus an almost entirely African-American population, as frailty is more common among African-American individuals [7]. The frailty index prevalence estimate was 1.4 times higher than the prevalence estimated by the frailty phenotype, which is in concordance with results found in the general population [15, 17–19]. Studies with large study samples of community-dwelling adults proposed that these two frailty models capture different but overlapping groups of older adults and that they cover different sides of the spectrum of frailty [20, 21]. In the community-dwelling population, the frailty index defines risk of adverse outcomes, including mortality, more precisely than the frailty phenotype does [18, 19]. Thus, the FI may be a promising risk indicator in the chronic care of pre-dialysis and dialysis population, but further prospective research with the frailty index in comparison with the more traditional vascular risk factors for adverse outcomes should be done in these populations.

In the current study population, age was not associated with frailty. Frailty is not only prevalent among patients aged above 65 years; more than a quarter of young or middle-aged participants were also identified to be frail. This emphasizes the view that ESRD as a chronic condition, through proposed mediators of accelerated decline in functioning, such as inflammation, oxidative stress and endocrinopathies could lead to frailty [22]. Similar to previous studies, female participants were more likely to be frail [17, 22–24]. The exact mechanism for this gender difference is unknown, but possible explanations could be a lower lean body mass and muscle strength in women leading to sarcopenia and frailty or the development of more frailty characteristics due to a longer life expectancy of women [5, 7, 17]. The association of a high Charlson comorbidity index score with frailty is consistent with the view on the development of frailty, as multiple stresses, e.g., multiple chronic diseases, could lead to decline in homeostatic reserve. Previous research showed that in the hemodialysis population not necessarily the number of comorbidities but more specific comorbid conditions, like diabetes mellitus or peripheral vascular disease, were associated with frailty [4, 8, 9, 25].

Strengths of this cross-sectional study included measurement of a validated construct of frailty. Due to the single-center study of 95 participants, limitations in statistical power to detect small subgroup effects and generalizability for the dialysis population have to be taken into account. In contrast to the limited number of included participants, there was a considerable number of non-responders. This could lead to an underestimation of the prevalence of frailty in this population. Modification of physical activity, as one of the components of the frailty phenotype, into a self-report single-question item could also have influenced the frailty phenotype prevalence estimate. By consensus, frailty is defined as a medical syndrome with multiple causes and contributors that is characterized by diminished strength, endurance and reduced physiologic function that increases an individual’s vulnerability for developing increased dependency and/or death. The clinical frailty scale might have been an alternative method for the adequate measurement of this clinical syndrome [26].

In this study, frailty according to the frailty index among young and old ESRD patients was high. As the population of dialysis and pre-dialysis patients is growing, frailty will become an important subject of clinical care. Because frailty is associated with poor clinical outcomes, falls, disability, hospitalization and mortality, it is important to identify those who are at high risk and are in need of comprehensive care in order to improve outcome for this vulnerable population [7, 15].

## Electronic supplementary material

Below is the link to the electronic supplementary material.

Supplementary material 1 (DOCX 20 kb)
