# Supplementary material for: High prevalence of frailty in end-stage renal disease
Source: Int Urol Nephrol. 2016 May 10;48:1357–62. doi: 10.1007/s11255-016-1306-z (PMC4949293; doi:10.1007/s11255-016-1306-z)
Supplement: Supplementary file 1 — Supplementary material 1 (DOCX 20 kb) [file 11255_2016_1306_MOESM1_ESM.docx]

## Supplementary material

## Table S1 Frailty assessment instruments

Characteristics Frailty assessment instruments, as used in this study.

| **Frailty Instrument** | **Components** | | **Measurement** | | | | **Classification** |
| --- | --- | --- | --- | --- | --- | --- | --- |
| **Frailty Index** | 38 items, each scored 0 - 1 | | | | | | Total score of positive items / Total available items = index score  Index score of ≥0.25: frail. |
|  | ADL/IADL | Help Bathing  Help Dressing  Help getting in/out of Chair  Help Walking around house  Help Eating  Help Grooming  Help Using Toilet  Help up/down Stairs  Help lifting 10 lbs  Help Shopping  Help with Housework  Help with meal Preparations  Help taking Medication  Help with Finances | | | Yes = 1, No = 0 | |  |
|  | Physical | - Lost more than 10 lbs in last year - Stayed in Bed at least half the day due to health (in last month) - Cut down on Usual Activity (in last month) | | | Yes = 1, No = 0 | |  |
|  |  | - Walk outside | | | <3 days = 1, ≤ 3 days = 0 | |  |
|  | Psychosocial | - Self Rating of Health | | | Poor = 1  Fair = 0.75  Good = 0.5  Very Good = 0.25  Excellent = 0 | |  |
|  |  | - How Health has changed in last year | | | Worse = 1  Better/Same = 0 | |  |
|  |  | - Feel Everything is an Effort - Feel Depressed - Feel Happy* - Feel Lonely - Have Trouble getting going | | | Most of time = 1  Some time = 0.5  Rarely = 0 | |  |
|  | Comorbidity | High blood pressure  Heart attack  Congestive Heart Failure  Stroke  Cancer  Diabetes  Arthritis  Chronic Lung Disease | | | Yes = 1  Suspect = 0.5  No = 0 | |  |
|  | Function test | MMSE | | <10 = 1  11–17 = 0.75  18–20 = 0.5  20–24 = 0.25  >24 =0 | | |  |
|  |  | Grip Strength (GS in kg) | | Men  GS ≤ cutoff =1  BMI ≤ 24, GS ≤ 29  BMI 24.1–28, GS ≤ 30  BMI >28, GS ≤ 32 | | Women  GS ≤ cutoff =1  BMI ≤ 23, GS ≤ 17  BMI 23.1–26, GS ≤ 17.3  BMI 26.1–29, GS ≤ 18  BMI>29, GS ≤ 21 |  |
|  |  | Body Mass Index (BMI) | | <18.5, ≥ 30 = 1  25-<30 = 0.5 | | |  |
|  |  | Rapid pace Walk, 20 feet | | >10 seconds =1 | | |  |
|  |  | Usual pace Walk, 20 feet | | >16 seconds =1 | | |  |
|  |  |  | | | | |  |

* This item was scored inversely: Most of time = 0; Some time = 0.5; Rarely = 1

## Table S1, continued.

Characteristics Frailty assessment instruments, as used in this study.

| **Frailty Instrument** | **Components** | **Measurement** | | **Classification** |
| --- | --- | --- | --- | --- |
| **Frailty Phenotype** | 5 items, each scored 0 or 1 | | | Score range: 0 to 5  0: non-frail  1-2: pre-frail  ≥3: frail |
|  | Weight loss | *In the last year, have you lost more than 10 pounds unintentionally (i.e., not due to dieting or exercise)*  Yes = frail for weight loss criterion | |  |
|  | Exhaustion | *(a) I felt that everything I did was an effort;*  *(b) I could not get going.*  *The question is asked “How often in the last week did you feel this way?”*  *0= rarely or none of the time (1 day)*  *1= some or a little of the time (1–2 days)*  *2 = a moderate amount of the time (3–4 days)*  *3 = most of the time*  Subjects answering “2” or “3” to either of these questions are categorized as frail by the exhaustion criterion. | |  |
|  | Walk Time (WS) | *Stratified by gender and height*  *Men*  Height ≤ 173 cm  Height > 173 cm  *Women*  Height ≤ 159 cm  Height > 159 cm | *Cutoff for Time to Walk 15 feet criterion for frailty.* WS ≥ cutoff = 1  ≥7 seconds  ≥6 seconds  ≥7 seconds  ≥6 seconds |  |
|  | Grip Strength (GS) | *Stratified by gender and body mass index (BMI)*  *Men*  BMI ≤ 24  BMI 24.1–26  BMI 26.1–28  BMI > 28  *Women*  BMI ≤23  BMI 23.1–26  BMI 26.1–29  BMI > 29 | *Cutoff for grip strength (Kg) criterion for frailty.* GS ≤ cutoff = 1  ≤29  ≤30  ≤30  ≤32  ≤17  ≤17.3  ≤18  ≤21 |  |
|  | Physical Activity^26^ | *How often do you engage in activities that require a low or moderate level of energy such as gardening, cleaning the car, or going for a walk?*  1 = “More than once a week”  2 = “Once a week”  3 = “One to three times a month”  4 = “Hardly ever or never”  Participants were score low physical activity when they answer ‘one to three times a month’ or ‘hardly ever’ | |  |
